# What do oncologists need to know about biosimilar products?

**DOI:** 10.1186/s40880-016-0151-x

**Published:** 2016-10-13

**Authors:** Linda K. S. Leung, Kevin Mok, Calvin Liu, Stephen L. Chan

**Affiliations:** 1 State Key Laboratory in Oncology in South China, Department of Clinical Oncology, Sir YK Pao Center for Cancer, Hong Kong Cancer Institute and Prince of Wales Hospital, The Chinese University of Hong Kong, Shatin, Hong Kong, China; 2 Faculty of Medicine, The Chinese University of Hong Kong, Hong Kong, China

**Keywords:** Biosimilar products, Biologic products, Regulation, Oncology

## Abstract

Many biologic products have improved the outcomes of cancer patients, but the costs can substantially burden healthcare systems. Biosimilar products can potentially reduce drug costs and increase patient access to beneficial treatments. Approval of a biosimilar product relies on the demonstration of “comparability” or “no clinically meaningful differences” as compared to its reference biologic product. Biosimilar products for erythropoietin, granulocyte colony-stimulating factor, trastuzumab, and rituximab are already available, and the regulatory processes in various countries are constantly evolving. It is important that oncologists be familiar with the potential issues surrounding the clinical use of biosimilar products. In this review article, we provide background information about biosimilar products and their regulatory approval processes, followed by a discussion of individual biosimilar drugs.

## Background

Biologic therapies, such as the monoclonal antibodies (mAbs) trastuzumab and bevacizumab, have prolonged the survival of cancer patients, but their high costs have also resulted in substantial financial burdens being placed on individual patients and on healthcare systems as a whole [1]. Because the patents of many biologic products in oncology are set to expire (Table 1), many biosimilar products are being developed and will soon become available in global pharmaceutical markets. The lower costs of these biosimilar drugs compared with their reference biologics can help reduce cancer drug costs and potentially allow more patients to gain access to the drugs, thus improving patient outcomes.

**Table 1:** Key oncological biologics whose patents either have expired or will expire soon

Biologic (brand name)	Manufacturer	Estimated patent expiry (month and year)	
		United States	Europe
*Supportive agents*			
Filgrastim (Neupogen)	Amgen/Roche/Jassen	Expired	Expired
Pegfilgrastim (Neulasta)	Amgen	Expired	Aug 2017
Epoetin alfa (Emprex/Epogen/Procrit)	Amgen/Jassen	Expired	Expired
Darbepoetin alfa (Aranesp)	Amgen	May 2024	Jul 2016
*Monoclonal antibodies*			
Trastuzumab (Herceptin)	Genentech/Roche	Jun 2019	Expired
Rituximab (Rituxan/MabThera)	Roche	Sep 2016	Expired
Cetuximab (Erbitux)	Eli Lilly/Bristol-Myers Squibb/Merck KgaA	Expired	Expired
Bevacizumab (Avastin)	Genentech/Roche	Jul 2019	Jan 2022

“Biosimilar”, as the name implies, is a biologic that is similar to the licensed “reference” drug. Before a biosimilar is incorporated into the clinical management of patients, clinicians should determine whether, based on efficacy and safety, the biosimilar can be used “instead of” or “interchangeably with” the reference biologic. Regulatory authorities play an important role in the development of biosimilars, and medical societies help guide clinicians on the use of biosimilars [2–5]. Currently available and of most interest in the field of oncology are biosimilars of the supportive agents filgrastim and erythropoietin and the mAbs trastuzumab and rituximab. Bevacizumab and cetuximab are innovator drugs in oncology whose patents will expire in the next few years [6]. Recent surveys conducted in America, Europe, and Asia showed that most clinicians are not very familiar with biosimilars [5, 7, 8]. In this review, we provide information that oncologists need to know about this new category of medicine.

### What is a biosimilar?

“Biosimilar” [3, 4], “similar biotherapeutic product” [2], “follow-on biologic” [9], and “subsequent entry biologic” [10] are terms for a successor drug that has the same mechanism of action as the original biologic. The World Health Organization (WHO) defines biosimilar as “a biotherapeutic product which is similar in terms of quality, safety, and efficacy to an already licensed reference biotherapeutic product” [2]. Unlike simple generic medicines that are chemically synthesized, biologics are produced by a more complex manufacturing process, are derived from living cells or organisms, and consist of relatively large and complex molecules [11]. Any variations in the manufacturing condition can result in alterations in biological function, causing changes in efficacy; and/or induce an immune response (immunogenicity), leading to a new adverse reaction. There is certain degree of variability in the manufacturing process of biologics that exists even between different batches of the same product. The expiration of a biologic’s patent unveils only the primary amino sequence and the structure of the drug, not precise production information. The attempted replica can be considered only highly similar to the original biologic, not truly “generic.” Therefore, the criteria for obtaining regulatory approval of chemically derived generic drugs are inappropriate for biosimilars. Table 2 summarizes the differences between biosimilars and generic drugs.

**Table 2:** Comparisons between biosimilars and generics

Feature	Biosimilars	Generics
Definitions	*US FDA* A biosimilar is a biological product that is highly similar to a licensed reference biological product notwithstanding minor differences in clinically inactive components; and there are no clinically meaningful differences between the biological product and the reference product in terms of the safety, purity, and potency of the product	*US FDA* A generic drug is identical to a brand name drug in dosage, safety, strength, route of administration, quality, performance, and intended use
	*EU EMA* A biosimilar is a biological medicinal product that contains a version of the active substance of an already authorized original biological medicinal product (reference medicinal product). Similarity to the reference medicinal product in terms of quality characteristics, biological activity, safety, and efficacy based on a comprehensive comparability exercise need to be established	*EU EMA* A generic medicine is a medicine that is developed to be the same as a medicine that has already been authorized (the “reference medicine”). It contains the same active substance(s) as the reference medicine, and it is used at the same dose(s) to treat the same disease(s) as the reference medicine
Manufacturing processes	Complex, as they are derived from living cells or organisms, sensitive to manufacturing changes	Simple, as produced by chemical synthesis
Immunogenicity	Immunogenic	Mostly non-immunogenic
Compared with original counterparts	Similar but not identical Need to demonstrate comparability (i.e., no clinically meaningful differences) to its comparator (the reference drug) Automatic substitution not recommended (some biosimilars might not carry all the same indications, especially if the reference biologics have multiple mechanisms of action)	Identical Therapeutically equivalent Allow automatic substitution

*US* the United States; *FDA* the Food and Drug Administration; *EU* the European Union; *EMA* the European Medicines Agency

### Biosimilar regulatory approval

The European Medicines Agency (EMA) is the body responsible for approving biosimilars in the European Union (EU), and it established the first legislative pathway for doing so. In 2005, it published guidelines governing the development of biosimilars; since then, it has developed individual guidelines for the biosimilars of granulocyte colony-stimulating factor (G-CSF), erythropoietin, and various mAbs [3]. The EMA defines biosimilars as “a biological medicinal product that contains a version of the active substance of an already authorized reference medicinal product and similarity to the reference product in terms of quality characteristics, biological activity, safety and efficacy based on a comprehensive comparability exercise needs to be established” [3]. The concept of “comparability” in reference to the original biologic is considered fundamental to the approval of a biosimilar. Comprehensive comparability studies should demonstrate similarity in physiochemical, biological, and immunological characteristics and in efficacy and safety. Immunogenicity has been a safety concern for biosimilars; thus, a robust pharmacovigilance system and risk management procedures should be in place to ensure long-term safety.

In the EU, the first biosimilar product was approved in 2006 [12]. Many countries soon followed the EMA’s lead. Since 2008, Australia has followed the EU guidelines and approved its first biosimilar in 2010 [13]. In 2013, the Australia’s Therapeutic Goods Administration released a guidance document on the evaluation of biosimilars [14]. Canada also follows the EU regulatory process and finalized its guidelines in 2010 [10]. In 2009, the WHO formalized guidelines on the evaluation of similar biotherapeutic products [2]. The United States of America (USA) has lagged behind in the development of biosimilars, having approved its first biosimilar only in March 2015 [9]. The United States Food and Drug Administration (US FDA) issued final guidelines in April 2015 [4]. It defines biosimilar as “a biological product that is highly similar to the reference product notwithstanding minor differences in clinically inactive components and that there are no clinically meaningful differences between the biological product and the reference product in terms of safety, purity and potency.” Emerging drug markets of Asia are typically generics driven; they offer an attractive market for the developers of biosimilars, but regulations vary across countries, and not every country has issued guidelines [15–21] (Table 3). Some Asian countries follow a specific biosimilar pathway that is akin to the EMA model [16–19, 22]. Singapore has approved biosimilar products only if they have been approved by other agencies namely, the EMA, the US FDA, the Australian Therapeutic Goods Administration, or the Health Canada [19]. In India, less stringent regulatory requirements have led to many biosimilars being available in the market since 2000; however, not until 2012 were guidelines issued [21]. In China, specific guidelines to be developed by the China Food and Drug Administration are pending; currently, the approval of biosimilars follows a simplified new product approval pathway [20]. Worldwide, regulations are evolving, and global harmonization of regulatory standards would definitely help manufacturers develop biosimilars in a more cost-effective way [23].

**Table 3:** Global regulations for the evaluation and approval of biosimilars

Feature	Yes	No
Own biosimilar guideline issued?	EU—EMA, 2005 Australia—TGA, 2013 Malaysia—NPCB, 2008 Singapore—HSA, 2011 Japan—PMDA, 2009 South Korea—MFDS, 2010 Canada—Health Canada, 2010 US—FDA, 2015 India—Ministry of Science and Technology and Ministry of Health and Family Welfare, 2012	New Zealand—Medsafe (but refers to both EU and US guidelines) China—CFDA (follows a simplified new product approval pathway)
Reference product must be registered in the region/country?	EU Australia Singapore Japan South Korea US India	Canada Malaysia (products registered in the reference countries Australia, Canada, EU, United Kingdom, France, Japan, Sweden, Switzerland, and US are considered acceptable)
Interchangeability designation?	US Singapore and Malaysia (allow if both biosimilar and reference product approved for the same indication but cannot be substituted with one another during a treatment period)	Europe Australia Japan South Korea Canada India
Extrapolation from one indication to another?	EU Australia Japan South Korea Singapore Malaysia Canada US India (allow for extrapolation of therapeutic similarity across indications in certain cases, depending on clinical experience, available literature data, and whether the same mechanisms of action or the same receptor(s) are involved in all indications)	

*EU* the European Union; *EMA* the European Medicines Agency; *TGA* the Therapeutic Goods Administration; *CDE* the Center for Drug Evaluation; *NPCB* the National Pharmaceutical Control Bureau; *HSA* the Health Sciences Authority; *PMDA* the Pharmaceuticals and Medical Devices Agency; *MFDS* the Ministry of Food and Drug Safety; *US* the United States; *FDA* the Food and Drug Administration; *Medsafe* the Medical Devices Safety Authority; *CFDA* the China Food and Drug Administration

**Fig. 1:**
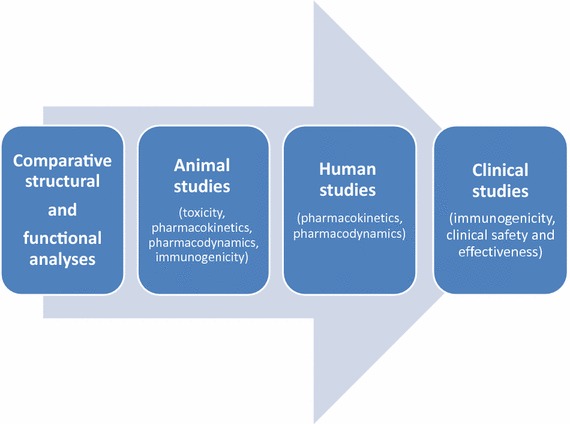
Stepwise approach to demonstrating biosimilarity between biosimilars and reference biologics

Overall, the USA and the EU have similar basic principles in terms of quality and clinical and non-clinical comparability testing strategies [3, 4]. A single reference product as comparator should be used throughout the studies to enable the generation of coherent data and conclusions. The dosage and the form and route of administration of the biosimilar and the reference biologic should be the same. The totality-of-the-evidence approach is adopted to review the marketing authorization application of biosimilar products, and a stepwise approach for demonstrating biosimilarity is recommended (Fig. 1). At each step, the biosimilar developer should evaluate and justify any identified uncertainties or differences between the biosimilar and the reference biologic and maintain an active dialogue with the approval agency, all of which affect the type and amount of data required to move on to the next step. Demonstrating high molecular similarity between the biosimilar and the reference biologic at the beginning reduces the amount of preclinical and clinical data that are subsequently required. If the reference biologic is licensed for more than one indication, similarity of the biosimilar should be demonstrated separately for each indication. Extrapolation of efficacy and safety data from one therapeutic indication to another is allowed, provided that there is evidence that the mechanisms of action and safety in the subpopulations evaluated are very likely to be equivalent. Currently, the EMA and the US FDA are collaborating to facilitate the global development of biosimilars. Regular meetings are being held between the two agencies to exchange information on the development of biosimilar products and on pharmacovigilance.

### Pharmacovigilance of biosimilars

Pharmacovigilance is “the science and activities relating to the detection, assessment, understand and prevention of adverse effects or any other drug related problem” [24]. Pharmacovigilance is particularly important when dealing with biologics because safety data from pre-authorization clinical studies only identify some potential risks and are insufficient to detect rare adverse events. Immunogenicity can be related to the route of administration, dosing regimen, patient-related factors, and disease-related factors, and it is an ongoing safety concern [3]. The case of erythropoietin antibody-mediated pure red cell aplasia (PRCA) is a good example of immunogenicity being identified by post-marketing surveillance, when a small change in the formulation of the biologic led to an adverse immune response [25]. In addition to immunogenicity, other safety concerns, such as the administration of hematopoietic colony-stimulating factors to healthy donors, which led to the development of hematologic malignancies, have been observed with biologics [26]. To ensure long-term safety, biosimilars are required to follow the same pharmacovigilance regulations as their reference counterparts. Required as part of the marketing application are a risk management plan describing the safety profile of the drug as well as proposed pharmacovigilance and risk minimization measures. After biosimilars are approved, companies are required to submit periodic safety reports. Prescribers should report any suspected serious adverse reactions associated with the use of biosimilars, and ensuring traceability of the biosimilars associated with adverse events is essential. To date, no specific safety concerns regarding approved and marketed biosimilars have been identified.

### Interchangeability between a biologic and its biosimilar

Generic medications can be used interchangeably with their branded originators since they are considered therapeutically equivalent; often, pharmacists may substitute a prescribed drug for a generic medication without the prior consent of the treating physician (known as “automatic substitution”) [23]. Interchangeability refers to switching back and forth between two medicinal products without any observed changes in efficacy or safety risk. Biosimilarity does not imply interchangeability, and interchangeability does not always imply substitutability.

Some biosimilars might not have all the same indications that the reference biologics are approved for, especially if the reference biologics have multiple mechanisms of action. Even if a biosimilar is approved for the same indication as the reference originator, it is generally recommended that automatic substitution not be allowed because the clinician should be fully aware of which drug is given to the patient. If, during a treatment period, alternation or switching between the biosimilar and the branded biologic cannot be avoided, this must be recorded accurately; maintaining pharmacovigilance by clearly delineating between biosimilars and branded originators is important.

The USA allows an “interchangeable” designation for biosimilars, provided that, in any given patient, the biosimilar can be expected to produce the same clinical results as the reference product and that the safety and efficacy observed when alternating or switching between the two remain the same [4]. Other countries, such as Australia and Canada, do not provide recommendations on whether a biosimilar can be used interchangeably with its reference medicine, and they have officially prohibited the automatic substitution of biologics [10, 14]. The EMA also does not provide an interchangeable recommendation when approving a biosimilar [3]. Individual EU countries must decide on the interchangeability between a biologic and its biosimilar [27].

### Biosimilar epoetins

Erythropoiesis-stimulating agents (ESAs), such as epoetin alfa (Eprex, Erypro), epoetin beta (NeoRecormon), and darbepoetin alfa (Aranesp), are approved for cancer patients with chemotherapy-induced anemia [28, 29]. They are considered equivalent in terms of efficacy and safety. In 2010, concerns about an increased risk of venous thromboembolism and an increased mortality risk associated with the use of ESAs by cancer patients led the European Society for Medical Oncology and the American Society of Clinical Oncology/American Society of Hematology to issue revised guidelines [29]. These guidelines recommended against the use of ESAs for the treatment of malignancy-associated anemia in patients not receiving concurrent myelosuppressive chemotherapy; they also recommended against the use of ESAs for patients receiving chemotherapy for curative intent, until further safety data are collected. However, the EMA’s Committee for Medicinal Products for Human Use stated that the benefits of using ESAs for approved indications (hemoglobin target range of 10–12 g/dL in chemotherapy-induced anemia) continue to outweigh the associated risks, including increased risk of tumor progression and venous thromboembolism and reduced survival, except for those cancer patients with a reasonably long life expectancy whose anemia should be treated by blood transfusions [30].

In 2007, two biosimilar epoetins–epoetin alfa, marketed as Binocrit, Abseamed, and Epoetin Alfa Hexal; and epoetin zeta, marketed as Retacrit and Silapo were approved in Europe [31–35] (Table 4). Comparability of these biosimilar epoetins with their reference drugs Eprex and Erypo was demonstrated in the setting of renal anemia [36]. Their use in the treatment of chemotherapy-induced anemia in cancer patients was approved because, based on the extrapolation of data, the mechanisms of action for epoetins are the same for all approved indications. PRCA caused by cross-reacting neutralizing antibodies against erythropoietin is a rare but known serious adverse event that has been observed with the use of erythropoietin in patients with chronic renal failure [37]. Therefore, the post-marketing and risk management plans of biosimilar epoetins particularly focus on PRCA.

**Table 4:** List of currently approved erythropoietin biosimilars

Region	Active substance (laboratory code)	Brand name (pharmaceutical company)	Indications in oncology	Approval date
Europe	Epoetin alfa (HX575)	Binocrit (Sandoz)	Treatment of anemia and reduction of transfusion requirements in adult patients receiving chemotherapy for solid tumors, malignant lymphoma, or multiple myeloma, and at risk of transfusion as assessed by the patient’s general status (e.g., cardiovascular status, pre-existing anemia at the start of chemotherapy)	28/08/2007
		Abseamed (Medice Arzneimittel)		28/08/2007
		Epoetin alfa Hexal (Hexal)		28/08/2007
	Epoetin zeta (SB309)	Retacrit (Hospira)		18/12/2007
		Silapo (Stada Arzneimittel)		18/12/2007
Australia	Epoetin lambda	Aczicrit (Sandoz/Novartis)	Treatment of anemia in patients with non-myeloid malignancies where anemia develops as a result of concomitantly administered chemotherapy and where blood transfusion is not considered appropriate	27/01/2010
		Grandicrit (Sandoz/Novartis)		27/01/2010
		Novicrit (Sandoz/Novartis)		27/01/2010
New Zealand	Epoetin alpha (HX575)	Binocrit (Novartis)	Treatment of anemia in patients with non-myeloid malignancies where anemia is due to the effect of concomitantly administered chemotherapy	27/02/2013

### Biosimilar G-CSF

G-CSF is widely used in the field of oncology [38]. It is used to decrease the incidence of chemotherapy-related febrile neutropenia, facilitate dose-intensity chemotherapy, and mobilize hematopoietic stem cells for collection. Currently, there are eight EMA-licensed filgrastim biosimilars, the first of which was licensed in Europe in 2008 [39–46]. These G-CSF biosimilars were approved by the EMA for all the registered indications of the reference drug Neupogen, based on their comparable efficacy and safety profiles in the treatment of chemotherapy-induced neutropenia [47–51] (Table 5). In March 2015, the US FDA approved its first biosimilar product Zarxio (filgrastim-sndz), which has all the same indications as the reference drug Neupogen [52]. Key studies showing the comparability in efficacy and safety of the biosimilar filgrastim to its reference drug are summarized in Table 6.

**Table 5:** List of currently approved granulocyte colony-stimulating factor biosimilars

Region	Active substance	Brand name (pharmaceutical company)	Indications in oncology	Approval date
Europe	Filgrastim-XM02	Ratiograstim (Ratiopharm)	Reduction in the duration of chemotherapy-induced neutropenia and the incidence of febrile neutropenia (except for chronic myeloid leukemia and myelodysplastic syndromes) Reduction in the duration of neutropenia caused by myeloablative therapy followed by bone marrow transplant Mobilization of peripheral blood progenitor cells	15/09/2008
		Tevagrastim (Teva)		15/09/2008
		Biograstim (ABZ-Pharma)		15/09/2008
	Filgrastim-EP2006	Filgrastim Hexal (Hexal)		06/02/2009
		Zarzio (Sandoz)		06/02/2009
	Filgrastim-PLD108	Nivestim (Hospira)		08/06/2010
	Apo-Filgrastim	Grastofil (Apotex)		18/10/2013
		Accofil (Accord)		18/09/2014
United States	Filgrastim-EP2006	Zarxio/(Sandoz) [Placeholder non-proprietary name: filgrastim-sndz]	Decrease in the incidence of infection, as manifested by febrile neutropenia, in patients with non-myeloid malignancies receiving myelosuppressive anti-cancer drugs associated with a significant incidence of severe neutropenia with fever Reduction in the time to neutrophil recovery and the duration of fever, following induction or consolidation chemotherapy treatment of patients with acute myeloid leukemia Reduction in the duration of neutropenia and neutropenia related clinical sequelae (e.g., febrile neutropenia) in patients with non-myeloid malignancies undergoing myeloablative chemotherapy followed by bone marrow transplantation Mobilization of autologous hematopoietic progenitor cells into the peripheral blood for collection by leukapheresis	06/03/2015
Australia	Filgrastim-PLD108	Nivestim (Hospira)	Decrease in the incidence of infection, as manifested by febrile neutropenia, in patients with non-myeloid malignancies receiving myelosuppressive anti-cancer drugs in doses not usually requiring bone marrow transplantation; Reduction in duration of neutropenia and clinical sequelae in patients undergoing induction and consolidation chemotherapy for acute myeloid leukemia; Mobilization of autologous peripheral blood progenitor cells alone, or following myelosuppressive chemotherapy, to accelerate neutrophil and platelet recovery by infusion of such cells after myeloablative or myelosuppressive therapy in patients with non-myeloid malignancies; Mobilization of peripheral blood progenitor cells in normal volunteers; for use in allogeneic peripheral blood progenitor cell transplantation; Treatment of patients receiving myeloablative chemotherapy, for reducing the duration of neutropenia and clinical sequelae following autologous or allogeneic bone marrow transplantation	16/09/2010
	Filgrastim-XM02	Tevagrastim (Aspen Pharmacare)		29/08/2011
	Filgrastim-EP2006	Zarzio (Sandoz)		07/05/2013
New Zealand	Filgrastim-PLD108	Nivestim (Hospira)	Mobilization of autologous peripheral blood progenitor cells alone, or following myelosuppressive chemotherapy and the mobilization of peripheral blood progenitor cells in normal donors [allogeneic peripheral blood progenitor cell (PBPC)] Reduction in the duration of neutropenia and the incidence of febrile neutropenia in patients treated with established cytotoxic chemotherapy for malignancy (with the exception of chronic myeloid leukemia and myelodysplastic syndromes) and for the reduction in the duration of neutropenia and its clinical sequelae in patients undergoing myeloablative therapy followed by bone marrow transplantation considered to be at increased risk of prolonged severe neutropenia	24/05/2012
	Filgrastim-EP2006	Zarzio (Novartis)		31/03/2014
Singapore	Filgrastim-PLD108	Nivestim (Hospira)	Reduction in the duration of neutropenia and the incidence of febrile neutropenia in patients treated with established cytotoxic chemotherapy for malignancy (with the exception of chronic myeloid leukemia and myelodysplastic syndromes) Reduction in the duration of neutropenia in patients undergoing myeloablative therapy followed by bone marrow transplantation considered to be at increased risk of prolonged severe neutropenia	17/07/2012
Japan	Filgrastim	Filgrastim BS (Mochida/Fuji)	Treatment in neutropenia induced by anti-cancer chemotherapy	21/11/2012
		Filgrastim BS (Nippon Kayaku/Teva)		28/02/2013
		Filgrastim BS (Sandoz)		24/03/2014

**Table 6:** Key studies of biosimilar filgrastim that demonstrated clinical equivalence to the reference drug

Active substance	Brand name	Key studies of biosimilar filgrastim demonstrating clinical equivalence to reference drug
Filgrastim-XM02	Ratiograstim/Tevagrastim/Biograstim	Multinational, multicenter, randomized, controlled phase III study; breast cancer patients receiving docetaxel/doxorubicin chemotherapy (*n* = 348) Reference drug, Neupogen [47] Multinational, multicenter, randomized, controlled phase III study; lung cancer patients receiving platinum based chemotherapy (*n* = 240) Reference drug, Neupogen [48] Multinational, multicenter, randomized, controlled phase III study; patients with non-Hodgkin lymphoma receiving chemotherapy (*n* = 92) Reference drug, Neupogen [49]
Filgrastim-EP2006	Filgrastim Hexal/Zarzio	Multinational, multicenter, randomized, controlled phase III study in chemo-naïve breast cancer patients receiving neoadjuvant/adjuvant docetaxel, doxorubicin, and cyclophosphamide chemotherapy (*n* = 218) Reference product, Neupogen [50]
Filgrastim-PLD108	Nivestim	Multicenter, randomized, controlled phase III study; breast cancer patients treated with doxorubicin and docetaxel in neoadjuvant/adjuvant or first-line metastatic setting (*n* = 279) Reference drug, Neupogen [51]

*PK* pharmacokinetics; *PD* pharmacodynamics

Enhanced by a 20-kDa polyethylene glycol molecule compared with filgrastim, pegfilgrastim has a longer half-life and is administered less frequently [53]. Pegfilgrastim has been approved by the US FDA and the EMA since 2002 and is indicated in non-myeloid cancer patients undergoing chemotherapy to decrease the incidence of febrile neutropenia [54, 55]. Unlike filgrastim, pegfilgrastim is not approved for hematopoietic progenitor cell mobilization. Since 2007, four pegfilgrastim biosimilars have been approved in India [56]. In December 2014, Apotex, in conjunction with Intas Pharmaceuticals, successfully filed its biosimilar pegylated apofilgrastim with the US FDA. Another biosimilar pegfilgrastim, LA-EP2006, developed by Sandoz, is being compared with its reference product Neulasta in three phase III studies; the results will be used to support the company’s registration application in the USA and the EU. The US patent for pegfilgrastim expired in October 2015; the EU patent is expected to expire in August 2017.

### Biosimilar trastuzumab

Trastuzumab is a humanized mAb that targets human epidermal growth factor receptor 2 (HER2). For patients with HER2-positive breast cancer, it is the standard of care in the neoadjuvant, adjuvant, and palliative settings [57, 58]. It is also indicated for the palliative treatment of HER2-positive gastric cancer. Biosimilar trastuzumab is the first biosimilar mAb available for the treatment of cancer (Table 7). In November 2013, the trastuzumab biosimilar Hertraz was launched in India after approval was granted by the Drug Controller General of India [59]. In January 2014, the South Korean Ministry of Food and Drug Safety approved the trastuzumab biosimilar CT-P6 (Herzuma) based on the results of global clinical trials involving 558 patients in 18 countries and 115 sites [60]. In January 2016, the Ministry of Health of the Russian Federation approved the trastuzumab biosimilar HERtiCAD based on the results of a randomized, multicenter clinical study (NCT01764022) comparing the pharmacokinetics, immunogenicity, safety, and efficacy of BCD-022 with those of the reference drug Herceptin, both in combination with paclitaxel in treating patients with metastatic breast cancer [61]. The study demonstrated the non-inferiority of BCD-022 compared with Herceptin in terms of efficacy and safety. The primary patent on Herceptin in Europe expired in July 2014. Biosimilar trastuzumab has not yet been approved in the EU, but several are in phase III development.

**Table 7:** List of currently approved biosimilar monoclonal antibodies in oncology

Region	Active substance	Brand name (pharmaceutical company)	Approved indications in oncology	Approval date
India	Trastuzumab	CANmab (Biocon)/Hertraz (Mylan)	HER2-positive breast cancer	25/11/2013
South Korea	Trastuzumab	Herzuma (Celltrion)	HER2-positive breast cancer Advanced (metastatic) gastric cancer	15/01/2014
Russia	Trastuzumab	HERtiCAD (Biocad)	Breast cancer	20/01/2016
Russia	Rituximab	AcellBia (CSJC biocad)	CD20-positive non-Hodgkin’s B-cell lymphoma	25/05/2014

*HER2* human epidermal growth factor receptor 2

### Biosimilar rituximab

Rituximab is an mAb that acts against CD20 to treat non-Hodgkin’s lymphoma (NHL) and chronic lymphocytic leukemia. Marketed by Roche as Rituxan and MabThera, rituximab was originally approved for use in 1997 in the USA [62] and in 1998 in the EU [63]. In November 2013, the patent for rituximab expired in the EU; in September 2016, it will expire in the USA. In 2007, Dr. Reddy’s Laboratories marketed Reditux in India as a biosimilar of rituximab to treat diffuse large B cell lymphoma [64]. However, Reditux is not a true biosimilar because it has not been studied head to head against its reference product. In May 2014, Biocad’s Acellbia (also known as BCD020), a biosimilar of rituximab, was approved in Russia for the treatment of NHL [65] (Table 6). The approval was based on the results of a randomized, open-labelled study, involving over 30 centers in Ukraine, Russia, and India, which showed that the drug’s pharmacokinetics, pharmacodynamics, efficacy, and safety were similar to rituximab [66].

## Conclusions

The rationale for the development of biosimilar products is to improve patient access to biological therapies in a safe and cost-effective way. As the technology advances and evolves, so too do regulations governing the development of biosimilars. Oncologists should be aware that biosimilars are not generics of biological medicines, but they do have comparable efficacy and safety to the originators. Pharmacovigilance is critical to ensuring the long-term quality and safety of biosimilars. With biosimilars, it is important to be mindful of concerns about drug substitutions and extrapolation across indications.
